# Differential Diagnosis and Molecular Stratification of Gastrointestinal Stromal Tumors on CT Images Using a Radiomics Approach

**DOI:** 10.1007/s10278-022-00590-2

**Published:** 2022-01-27

**Authors:** Martijn P. A. Starmans, Milea J. M. Timbergen, Melissa Vos, Michel Renckens, Dirk J. Grünhagen, Geert J. L. H. van Leenders, Roy S. Dwarkasing, François E. J. A. Willemssen, Wiro J. Niessen, Cornelis Verhoef, Stefan Sleijfer, Jacob J. Visser, Stefan Klein

**Affiliations:** 1 Department of Radiology and Nuclear Medicine, Erasmus Medical Center, Rotterdam, The Netherlands; 2 Department of Medical Informatics, Erasmus Medical Center, Rotterdam, The Netherlands; 3 Department of Surgical Oncology, Erasmus MC Cancer Institute, Erasmus Medical Center, Rotterdam, The Netherlands; 4 Department of Medical Oncology, Erasmus MC Cancer Institute, Erasmus Medical Center, Rotterdam, The Netherlands; 5 Department of Pathology, Erasmus Medical Center, Rotterdam, The Netherlands; 6 Faculty of Applied Sciences, Delft University of Technology, Delft, The Netherlands

**Keywords:** Gastrointestinal stromal tumors, Sarcoma, Machine learning, Tomography, X-ray computed, Radiomics

## Abstract

Treatment planning of gastrointestinal stromal tumors (GISTs) includes distinguishing GISTs from other intra-abdominal tumors and GISTs’ molecular analysis. The aim of this study was to evaluate radiomics for distinguishing GISTs from other intra-abdominal tumors, and in GISTs, predict the *c-KIT*, *PDGFRA*, *BRAF* mutational status, and mitotic index (MI). Patients diagnosed at the Erasmus MC between 2004 and 2017, with GIST or non-GIST intra-abdominal tumors and a contrast-enhanced venous-phase CT, were retrospectively included. Tumors were segmented, from which 564 image features were extracted. Prediction models were constructed using a combination of machine learning approaches. The evaluation was performed in a 100 × random-split cross-validation. Model performance was compared to that of three radiologists. One hundred twenty-five GISTs and 122 non-GISTs were included. The GIST vs. non-GIST radiomics model had a mean area under the curve (AUC) of 0.77. Three radiologists had an AUC of 0.69, 0.76, and 0.84, respectively. The radiomics model had an AUC of 0.52 for *c-KIT*, 0.56 for *c-KIT *exon 11, and 0.52 for the MI. The numbers of *PDGFRA*, *BRAF*, and other *c-KIT* mutations were too low for analysis. Our radiomics model was able to distinguish GISTs from non-GISTs with a performance similar to three radiologists, but less observer dependent. Therefore, it may aid in the early diagnosis of GIST, facilitating rapid referral to specialized treatment centers. As the model was not able to predict any genetic or molecular features, it cannot aid in treatment planning yet.

**Supplementary Information:**

The online version contains supplementary material available at 10.1007/s10278-022-00590-2.

## Introduction

Gastrointestinal stromal tumors (GISTs) are rare mesenchymal tumors of the gastrointestinal tract, with an estimated incidence between 10 and 15 cases per million persons per year [1, 2]. The most common tumor locations are the stomach (56%) and the small intestine (32%) [2]. Differentiating GISTs from other intra-abdominal tumors (non-GISTs) is highly important for early diagnosis and treatment planning [3]. Due to the rarity of GISTs, establishing the correct diagnosis can be challenging. Computed tomography (CT) is the imaging modality of choice in GIST diagnosis [4], but assessment through an invasive tissue biopsy is generally required [5]. A non-invasive and quicker method may aid in the early assessment of GISTs, allowing rapid transfer of such patients to specialized treatment centers.

Treatment planning of GISTs is based on their molecular profile. The mitotic index (MI) reflects the proliferative rate of GISTs, correlates with survival and risk of metastatic spread [6], and determines the use of adjuvant systemic treatment. Treatment decisions are also based on the GISTs’ mutational status. *PDGFRA* exon 18 mutated (Asp842Val) GISTs are resistant to imatinib [7]. GISTs with a *c-KIT* exon 11 mutation have shown a greater sensitivity for imatinib than those with a *c-KIT* exon 9 mutations [3]. The MI and these genetic mutations are currently assessed through an invasive tissue biopsy. Prediction of such molecular characteristics based on imaging could guide treatment planning while awaiting the results of a final tissue biopsy.

Radiomics relates imaging features to molecular characteristics in order to contribute to diagnosis, prognosis, and treatment decisions. Radiomics has shown promising results in risk stratification of GISTs [8–17], but has not been used to distinguish GISTs from non-GISTs nor to predict the molecular profile.

The aim of this study was to evaluate whether radiomics based on CT is capable of (1) differentiating GISTs from other intra-abdominal tumors resembling GISTs prior to treatment, i.e., the differential diagnosis and (2) predicting the presence and type of mutation (*BRAF*, *PDGFRA*, and *c-KIT*) and the MI of GISTs, i.e., the molecular analysis, also called “radiogenomics”.

## Materials and Methods

### Data Collection

Approval by the Erasmus MC institutional review board was obtained (MEC-2017–1187). Patients from our institute between 2004 and 2017 with a histopathologically proven primary GIST or intra-abdominal tumors resembling GIST with at least a contrast-enhanced venous-phase CT prior to treatment [3, 18] were retrospectively included. The cohort of intra-abdominal tumors resembling GISTs was composed of consecutive intra-abdominal benign and malignant spindle cell and epithelioid non-GIST soft tissue tumors [5]. Age at diagnosis, sex, and tumor location (based on radiology reports) were collected. The sample sizes of the non-GIST and the GIST cohort were matched. The non-GIST subtypes were balanced, i.e., a similar number of patients per subtype was randomly included.

GISTs with a known mutation status and/or MI prior to therapy were included in the molecular analysis. Both were obtained from pathology reports. The mutation was categorized as “absent” or “present” for each type (e.g., *c-KIT*) and subtype (e.g., *c-KIT* exon 11). The MI (expressed in high power fields (HPF), magnification 40 × , totaling 5mm^2^), determined on biopsy or excision material, was split into low (≤ 5/50 HPF) and high (> 5/50 HPF) [19]. An adjusted MI was calculated per 50 HPF when the MI was not counted per 50 HPF. As not all of these characteristics may have all been analyzed in all patients, if a specific mutation (e.g., *c-KIT*) or the MI was not stated in the pathology reports, this was categorized as “missing” and the patient was not included in the related radiomics analysis.

### Radiomics

**Fig. 1:**
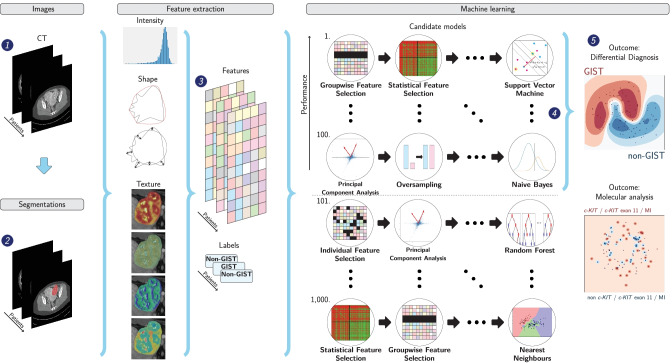
Schematic overview of the radiomics approach: adapted from Vos et al. [24]. Input to the algorithm are the CT images (1). Processing steps then include segmentation of the tumor (2), feature extraction (3), and the creation of machine learning decision models (5), using an ensemble of the best 100 workflows from 1000 candidate workflows (4), which are different combinations of the different processing and analysis steps (e.g., the classifier used). *Abbreviations: GIST, gastrointestinal stromal tumor; MI, mitotic index

Figure 1 depicts the radiomics workflow. Tumors were manually segmented once by one of two clinicians under the supervision of a musculoskeletal radiologist (5 years of experience) using in-house developed software [20]. A subset of 30 GISTs was segmented by both clinicians, in which inter-observer variability was evaluated through the pairwise Dice similarity coefficient (DSC), with a DSC > 0.70 indicating good agreement [21]. For each lesion, 564 features quantifying intensity, shape, and texture were extracted using the PREDICT [22] (version 3.1.13) and PyRadiomics [23] (version 3.0.1) toolboxes (see Supplemental Material 1). The WORC toolbox (version 3.4.0) was used to create a decision model from the features [24–26]. In WORC, radiomics is formulated as a modular workflow consisting of multiple components, e.g., feature selection, resampling, and machine learning. For each component, a variety of commonly used algorithms and their associated hyperparameters are included. Using automated machine learning, WORC automatically constructs and optimizes the radiomics workflow to determine which combination of algorithms and hyperparameters maximizes the prediction performance on the training set. The final model consists of an ensemble of the 100 workflows performing best on the training set (for details, see Supplemental Material 2). The code for the feature extraction and model creation has been published open source [27].

### Experimental Setup

Evaluation of all models was done through a 100 × random-split cross-validation. In each iteration, the data was randomly split into 80% for training and 20% for testing in a stratified manner (see Supplemental Fig. S1). Within the training set, the WORC model optimization was performed using an internal cross-validation (5 ×). Hence, all optimization was done on the training set to eliminate any risk of overfitting on the test set.

Performance was evaluated using the area under the curve (AUC) of the receiver operating characteristic (ROC) curve, balanced classification accuracy (BCA) [28], sensitivity, and specificity. The positive classes were defined as GIST, the presence of the mutations, and a high MI. The mean performance measures over the 100 cross-validation iterations were computed, and their 95% confidence intervals (CIs) were constructed using the corrected resampled *t*-test [29]. When plotting ROC curves, confidence bands were constructed using fixed-width bands [30].

First, to evaluate the predictive value of imaging, a radiomics model based on imaging only was evaluated. To assess the predictive value of volume alone, an additional volume-only model was trained. Second, as radiologists frequently use age, sex, and location in the differential diagnosis, an additional model for the differential diagnosis was created based on radiomics, age, sex, and location.

### Model Insight

The differences in feature values and CT acquisition parameters between the GIST and non-GIST cohorts were assessed using a Mann–Whitney *U* univariate statistical test for continuous variables, and a chi-square test for categorical variables. *P*-values of the features were corrected for multiple testing using the Bonferroni correction, i.e., multiplying the *p*-values by the number of tests. Values of *p* < 0.05 were considered statistically significant. For the statistically significant acquisition parameters, the individual predictive value was assessed using the AUC.

To gain insight into the models, the patients were ranked from typical to atypical for both the GIST and non-GIST groups, based on the consistency of the model predictions. This was determined by the number of times (percentage) that a patient was classified correctly when included in the test set of a cross-validation iteration. Typical examples for each class consisted of the patients who were always classified correctly; atypical vice versa.

The robustness of the radiomics features and model to variations in the segmentations and the CT acquisition protocols was evaluated using the intra-class correlation coefficient (ICC) [31, 32] and ComBat [33, 34] (see Supplemental Material 3).

### Performance of the Radiologists

To compare the models with clinical practice, three radiologists (5, 15, and 12 years of experience) independently scored the lesions on a ten-point scale to indicate their certainty of the tumor being a GIST (i.e., 1 = strongly disagree, 10 = strongly agree). The radiologists were blinded for the diagnosis but had access to the CT scan, patient age, and sex. The agreement between radiologists was evaluated using Cohen’s kappa. To enable direct statistical comparison, the radiomics model was evaluated in an additional leave-one-out cross-validation, after which the DeLong test was used to compare the AUCs [35].

## Results

### Dataset

The dataset included 247 patients (125 GISTs, 122 non-GISTs) (see Table 1) and has been publicly released [36]. The dataset of 247 CT scans originated from 66 different scanners, resulting in variation in the acquisition protocols. The scans originated from four different manufacturers (Siemens, Berlin, Germany: 126; Philips, Eindhoven, the Netherlands: 63; General Electric, Boston, United States: 10; Toshiba, Tokyo, Japan: 48). Between the GIST and non-GIST scans, statistically significant differences were found in peak kilovoltage (KVP) (*p* = 0.025), slice thickness (*p* = 9.52 × 10^−4^). Their individual predictive power was however low (AUC of 0.56 for KVP, 0.60 for slice thickness), which is supported by the inter-quartile ranges being the same in GISTs and non-GISTs (KVP: (100.0, 120.0), slice thickness: (3.0, 5.0)). No statistically significant differences were found in manufacturer (*p* = 0.15), pixel spacing (*p* = 0.10), or tube current (*p* = 0.15). On the subset of 30 GISTs that was segmented by both observers, the mean DSC was 0.84 (standard deviation of 0.20), indicating good agreement.

**Table 1:** Clinical and CT scan characteristics of the dataset. The dataset of 247 CT scans originated from 66 different scanners, resulting in variation in the acquisition protocols. Note that while the imaging characteristics are specified per tumor type, these do not identify separate scanners: patients of various tumor types are scanned on the same scanners

	GISTs	Schwannoma	Leiomyo-sarcoma	Leiomyoma	Esophageal/gastric junctional adenocarcinoma	Lymphoma
**Number**	125	22	25	25	25	25
**Sex**						
Male Female	66 (53%) 59 (47%)	11 (50%) 11 (50%)	7 (28%) 18 (72%)	6 (24%) 19 (76%)	16 (64%) 9 (36%)	18 (72%) 7 (28%)
**Age at diagnosis**^a^	64 (56–72)	59 (45–67)	60 (53–71)	49 (41–59)	65 (56–74)	62 (52–67)
**Tumor location**^b^						
(Distal) esophagus Stomach Small intestine Colon Rectum Pelvis Mesentery Uterus Other	- 80 (64%) 29 (23%) 1 (1%) 7 (6%) 1 (1%) - - 7 (6%)	- 2 (9.1%) - - - 7 (31.8%) - - 13 (59.1%)	- 1 (4%) 1 (4%) 2 (8%) - 5 (0%) - 2 (8%) 14 (56%)	6 (24%) 3 (12%) - - - 2 (8%) - 13 (52%) 1 (4%)	5 (20%) 20 (80%) - - - - - - -	- 2 (8%) 4 (16%) 1 (4%) - 1 (4%) 7 (28%) - 10 (40%)
**Tumor volume (cl)**^a^	15.7 (4.3–52.6)	13.9 (1.6–29.7)	12.9 (6.7–99.6)	8.2 (1.6–25.5)	1.6 (0.7–3.1)	9.4 (4.6–29.4)
**Acquisition protocol**						
Slice thickness (mm)^a,c^	5.0 (3.0–5.0)	5.0 (2.0–6.0)	5.0 (3.0–5.0)	3.0 (3.0–5.0)	4.0 (3.0–5.0)	3.0 (3.0–3.0)
Pixel spacing (mm)^a,c^	0.72 (0.68–0.78)	0.74 (0.68–0.79)	0.72 (0.68–0.78)	0.75 (0.68–0.84)	0.74 (0.66–0.78)	0.77 (0.69–0.85)
Tube current (mA)^a,c^	189 (129–283)	162 (115–206)	221 (160–349)	210 (147–395)	210 (142–312)	207 (145–301)
Peak kilovoltage^a,c^	120 (100–120)	120 (120–120)	120 (100–120)	120 (100–120)	120 (100–120)	100 (100–100)

*GIST* gastrointestinal stromal tumor, *cl* centiliter, *mm* millimeter, *mA* milliampere

^a^Median (inter-quartile range)

^b^Percentages may not add up to 100% because of rounding

^c^Other values than those given in the inter-quartile range do occur

Of the 125 GIST patients, two were not included in the molecular radiomics analysis as the molecular characteristics were obtained after receiving systemic treatment, resulting in 123 GIST patients included in the molecular analysis. The mutation analysis was performed on tissue obtained from the primary lesion, except for three patients where a metastatic hepatic lesion was used. *c-KIT* mutational analysis was performed in 98/123 (80%) GISTs. One patient had a *c-KIT* mutation which was not further specified. Twenty-six out of 98 patients (27%) had no *c-KIT* mutation. The majority of patients had a *c-KIT* exon 11 mutation (*N* = 59, 60%). Due to the low numbers of *c-KIT* exon 9 (*N* = 10), *c-KIT* exon 13 (*N* = 2), *PDGFRA* (*N* = 14), and *BRAF* (*N* = 0), these mutations were excluded from further analysis.

The MI was analyzed in 90/123 (73%) GISTs (55 low, 35 high). The MI of 33 (37%) GISTs was converted to the adjusted MI. The MI was determined on excision material in 54 (60%) patients, and on biopsy material in 36 (40%) patients, including one patient in which the MI was based on the hepatic GIST metastasis.

### Differential Diagnosis

**Fig. 2:**
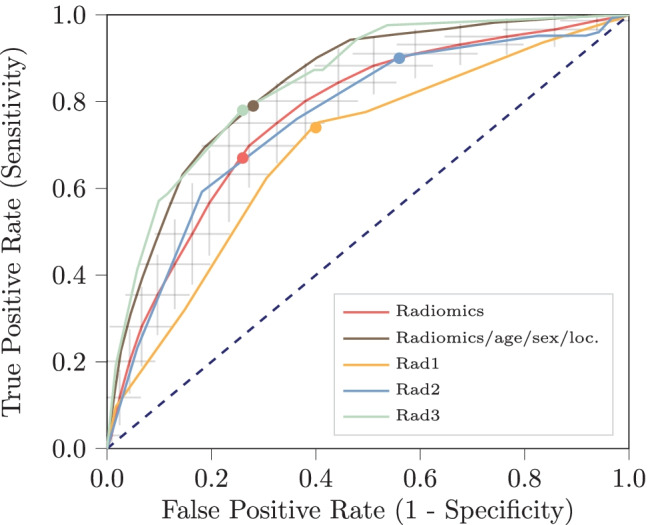
Receiver operating characteristic curves of the models for the differential diagnosis based on radiomics only and radiomics, age, sex, and tumor location. Additionally, the curves for scoring by three radiologists are shown, and the cutoff points for both the models and the radiologists. For the radiomics model based on imaging only, the grey crosses identify the 95% confidence intervals of the 100 × random-split cross-validation; the red curve is fit through their means

The performances of the models distinguishing GISTs from non-GISTs are shown in Table 2; the ROC curves are shown in Fig. 2. The radiomics model, i.e., based on imaging only, had a mean AUC of 0.77. An overview of the selected algorithms and hyperparameters for each cross-validation iteration in this model can be found online [27]. Only using volume did not perform well (AUC of 0.56). Combining radiomics with age, sex, and location yielded an improvement (AUC of 0.84).

**Table 2:** Performances of the models for the differential diagnosis based on radiomics features only, and radiomics, age, sex and tumor location, and that of the three radiologists (Rad1-3). Values for the models are the mean presented with their 95% confidence intervals

	Radiomics	Radiomics + age + sex + location	Rad1	Rad2	Rad3
**AUC**	0.77 [0.71, 0.83]	0.84 [0.79, 0.90]	0.69	0.76	0.84
**BCA**	0.70 [0.65, 0.76]	0.76 [0.70, 0.82]	0.67	0.67	0.76
**Sensitivity**	0.66 [0.56, 0.76]	0.79 [0.71, 0.88]	0.74	0.90	0.78
**Specificity**	0.74 [0.66, 0.83]	0.72 [0.61, 0.83]	0.60	0.44	0.74

*AUC* area under the receiver operating characteristic curve, *BCA* balanced classification accuracy, *Rad1, Rad2, and Rad3* radiologists 1, 2, and 3

The performance of the radiologists is shown in Table 2; their ROC curves are shown in Fig. 2. The three radiologists respectively had a lower (0.69), similar (0.76), and higher (0.84) AUC than the radiomics model. Compared to the model with the same inputs, i.e., based on radiomics, age, sex, and tumor location, the AUCs of the first two radiologists were lower, while the AUC of the third radiologist was similar. Cohen’s kappa measures between the pairs of radiologists were 0.20, 0.31, and 0.33, all indicating poor inter-observer agreement. The DeLong test between the pairs of radiologists indicated a statistically significant difference in performance for radiologists 1 versus 3 (*p* = 6 × 10^−5^) and 2 versus 3 (*p* = 0.01). The radiomics model evaluated in a leave-one-out cross-validation (AUC of 0.82) performed statistically significantly better than the first radiologist (*p* = 0.0018); for comparison with the other radiologists, the differences were not statistically significant.

### Evaluation of Models for the Molecular Analysis

For the *c-KIT* mutation stratification and MI predictions, the performance of the model based on radiomics, age, and sex is depicted in Table 3. All models had a mean AUC close to guessing (0.50) and focused on the majority class (*c-KIT* mutation and *c-KIT* exon 11 mutation: high sensitivity, low specificity; MI vice versa).

**Table 3:** Performance of the model based on radiomics, age, and sex, for the GIST mutation stratification and the mitotic index prediction. First column: *c-KIT* presence vs. absence; second column: *c-KIT* exon 11 presence vs. absence; third column: mitotic index (≤ 5/50 HPF vs. > 5/50 HPF). The number of patients included in each analysis (*N*) is mentioned in the heading. Values are presented with their 95% confidence intervals

	*c-KIT* (*N* = 98)	*c-KIT* exon 11 (*N* = 96)	Mitotic index (*N* = 90)
**AUC**	0.51 [0.36, 0.66]	0.57 [0.45, 0.68]	0.54 [0.42, 0.65]
**BCA**	0.49 [0.45, 0.54]	0.53 [0.44, 0.63]	0.51 [0.41, 0.60]
**Sensitivity**	0.96 [0.91, 1.0]	0.70 [0.54, 0.87]	0.27 [0.08, 0.46]
**Specificity**	0.03 [0.0, 0.11]	0.36 [0.20, 0.53]	0.75 [0.61, 0.88]

*AUC* area under the receiver operating characteristic curve, *BCA* balanced classification accuracy

### Model Insight

As the molecular analysis models did not perform well, the model insight analysis was only conducted for the differential diagnosis. The *p*-values of the feature importance analysis are shown in Supplemental Table S1. In total, 43 features had significant *p*-values after Bonferroni correction (1.1 × 10^−17^ to 4.6 × 10^−2^). These included the tumor location (1.1 × 10^−17^), two intensity features, three orientation features, four shape features of which three related to the tumor area, and 33 texture features. A list of these features and their *p*-values has been added to the mentioned published code [27]. Volume was not found to be significant.

**Fig. 3:**
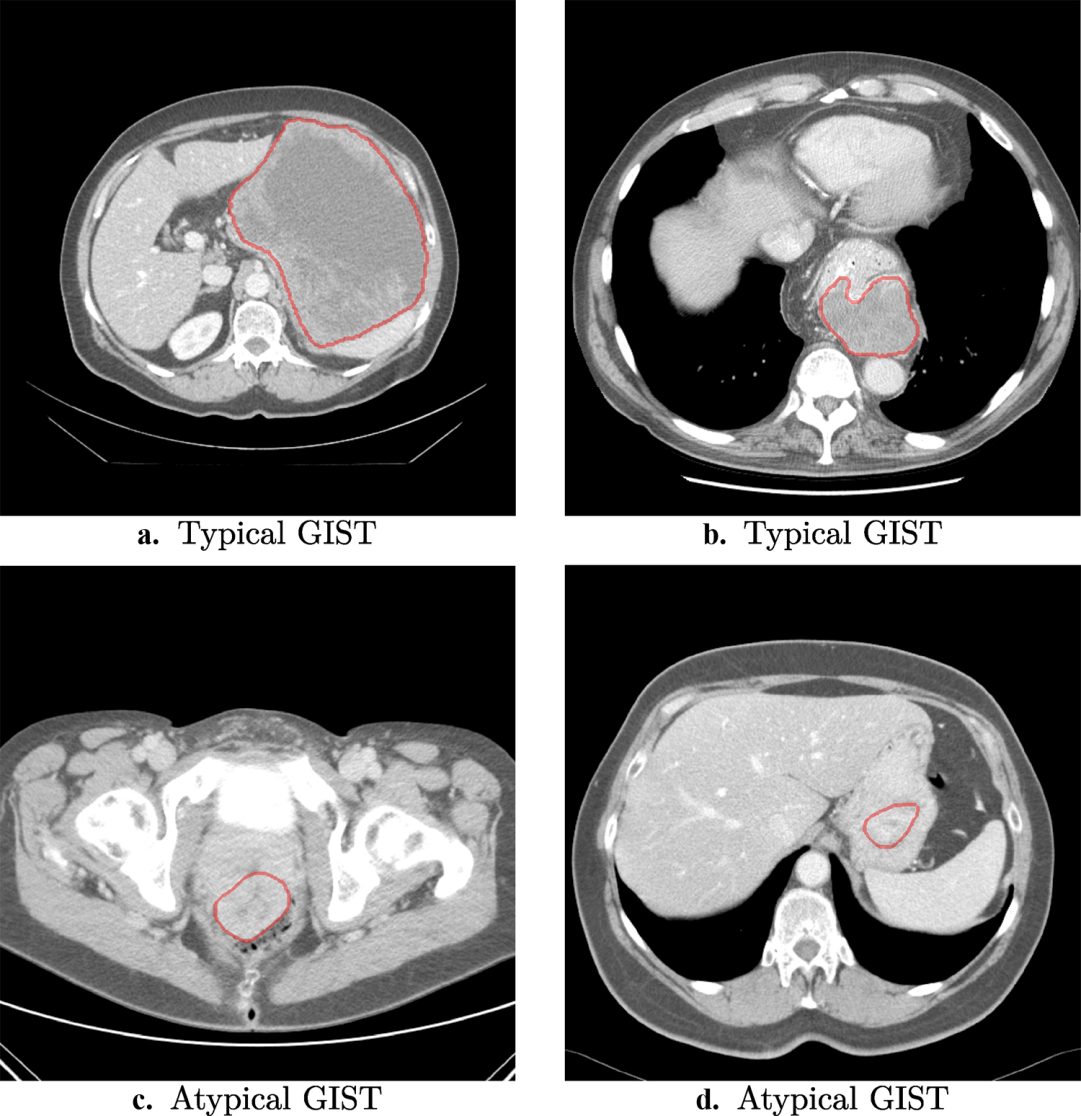
Examples of GISTs always correctly or always incorrectly classified by the radiomics model. The typical examples (**a** and **b**) are two of the GISTs always classified correctly by the radiomics model; the atypical examples (**c** and **d**) are two of the GISTs always classified incorrectly by the radiomics model

GISTs were ranked from typical to atypical as identified by the radiomics model. Of the 247 patients, 104 tumors (44 GISTs, 60 non-GISTs, 42%) were always classified correctly and were thus considered typical. Twenty-nine tumors (18 GISTs, 11 non-GISTs, 12%) were always classified incorrectly and thus atypical. In Fig. 3, four CT slices of such typical and atypical examples of GISTs are shown. Visual inspection of the tumors on imaging defined as typical or atypical by the radiomics model showed a relation with necrosis (more present in typical GIST, typically a necrotic core) and shape (more compact, circular, and non-lobulated for typical GIST). The tumors which were equally often classified as GIST and non-GIST in the cross-validation iterations were mostly small tumors. The typical imaging characteristics used by the model and the difficulty with small tumors correspond to findings in the literature on GIST risk stratification [14, 37]. Smaller tumors were also more often misclassified by the radiologists in our study.

Only using features with a good (ICC > 0.75, 327/564 features) or excellent (ICC > 0.9, 197/564 features) reliability across the segmentations lowered the performance (AUC of 0.67 for both). Using ComBat to harmonize the features for manufacturer or protocol differences yielded a similar performance as without (AUC of 0.80 and 0.77, respectively). Detailed results for these experiments are shown in Supplemental Table S2.

## Discussion

Radiomics can distinguish GISTs from other intra-abdominal tumors with a performance similar to three radiologists. Radiomics could not predict the presence and subtype of *c-KIT* mutations or the MI.

Diagnosing GISTs is currently done manually by radiologists and confirmed through a tissue biopsy [4, 38, 39]. The ability to distinguish rare GISTs from non-GISTs on routine CT scans through radiomics could be a quick method for the initial assessment of intra-abdominal tumors. Radiomics could aid quick referral of GIST patients from a peripheral hospital to a center of expertise, shortening time to diagnosis by refining patient selection prior to biopsies, and prevent GISTs from being missed (i.e., false negatives), unnecessary referral, or even treatment for non-GISTs (i.e., false positives). To our knowledge, this is the first study to evaluate the GIST differential diagnosis on many locations through an automated radiomics approach on a large, multi-scanner dataset, and compare the performance of the model with that of the radiologists.

There were significant performance differences between the radiologists, and their agreement was poor, indicating high observer dependence. The advantages of the radiomics model are that it is automatic and observer independent, assuming the segmentation is reproducible as indicated by the high DSC and that it will always give the same prediction on the same image, thereby improving consistency over manual scoring.

In clinical practice, tumor location is highly relevant for distinguishing GISTs from non-GISTs, as GISTs grow typically in the stomach or small intestines [2]. In our study, tumor location was based on radiology reports, which is subjective and occasionally fails to report the true tumor primary origin [19]. Moreover, the tumor location distribution in our dataset may not be a correct representation of the overall population, e.g., only non-GISTs were located in the uterus. Despite the subjectivity of potential bias in tumor location, we added location to the imaging model for a fair comparison with the radiologists. Although this led to a higher AUC, a model based on location, e.g., simply classifying all lesions in the uterus as non-GISTs, is unfeasible and cannot be applied in the general population. The radiomics model rather predicts the likelihood of a lesion being a GIST purely based on the imaging appearance. Further research on location-matched datasets is required to investigate the value of location in the GIST differential diagnosis model.

In the literature, radiomics for risk classification or outcomes such as malignant potential or aggressive behavior for GISTs [8–17] has mostly been based on criteria such as the Armed Forces Institute of Pathology criteria, modified National Institutes of Health consensus criteria of 2008, and the modified Fletcher classification system [3, 40–44]. These studies illustrate the clinical need for new methods to stratify GISTs and show the potential of radiomics for GISTs. Our first contribution with respect to the existing literature is the focus on the diagnostic trajectory of GISTs, to simplify the diagnostic process of this rare tumor type by predicting the differential diagnosis. Existing studies mainly focus on risk classification, which has a less apparent direct application in clinical practice, and generally first require the GIST differential diagnosis to be applicable [3, 40–43]. Second, our method determines the optimal radiomics pipeline from a large number of radiomics algorithms and parameters, automatically evaluating a large number of radiomics methods, whereas existing studies typically report the results of a “hand-crafted,” manually optimized radiomics pipeline [8–17]. Moreover, through an extensive cross-validation scheme, all model optimization was performed on the training dataset, eliminating the risk of overfitting the model on the test set. Lastly, we evaluated the model’s robustness to segmentation and scanner variations.

Our model was not able to distinguish different genetic mutations or the MI of GISTs, which may be attributed to various factors. First, the dataset for the mutation analysis was relatively small (e.g., 90 patients in the MI analysis), which may have been too small for radiomics to learn from. Second, the use of different gene panels for the GIST mutational analysis over the years may have resulted in inaccuracies in the golden standard. Additionally, this might have led to a potential underestimation of mutation prevalence in the current cohort, as newer sequencing techniques use larger gene panels and have a higher sensitivity. Third, other (more complex or deep learning based) radiomics methods may be required to discover more intricate features. Lastly, the negative results may simply suggest that molecular characteristics such as a *c-KIT* mutation are too subtle to detect solely based on portal venous phase CT imaging characteristics. Other CT phases or modalities (e.g., magnetic resonance imaging) could provide more useful information.

Our study has several limitations. First, there was heterogeneity in the acquisition protocols. There were two acquisition parameters (KVP and slice thickness) with statistically significant differences between GISTs and non-GISTs, but their individual predictive power was low. Hence, although a minor positive bias due to heterogeneity in acquisition protocols cannot be completely ruled out, the predictive performance cannot be attributed to this bias alone. Alternatively, this heterogeneity may have also negatively affected the performance. Nevertheless, the radiomics model achieved a promising performance, similar to three experienced radiologists, suggesting high generalizability. Second, complete histologic data was only available for a subset of the patients. No data regarding the clinical outcome such as survival or recurrence was available for the GISTs. Finally, the current radiomics approach requires manual segmentation. While accurate, this process is also time-consuming and potentially subject to observer variability, although the DSC indicated good agreement. Only using features with a good or excellent reliability across the segmentations lowered the performance. This may indicate that there are features that have a low reliability but a high predictive power, thus resulting in low performance when removing these. Alternatively, it may indicate overfitting of the model to observer-dependent characteristics of the segmentation and thus exploitation of a bias in the segmentations. Automatic segmentation methods may help to overcome this limitation.

Future work should focus on the extension of the dataset, leading to more statistical power, potentially improving the performance as the model has more cases to learn from, and paving the way for more data-driven approaches such as deep learning. Also, this may result in sufficient samples to study the prediction of less common GIST mutations. Next, external validation of our findings on an independent, external dataset is required. Eventually, this may be followed by a prospective clinical trial with harmonized acquisition protocols in which the performance, as well as the cost-effectiveness, is assessed.

## Conclusions

Our radiomics model was able to distinguish GIST from non-GIST intra-abdominal tumors based on pre-treatment CT imaging with a performance similar to three experienced radiologists, but is less observer dependent. Our model may therefore aid clinicians early on in the diagnostic chain to ensure rapid transfer of GISTs to specialized centers. The model was not able to predict the *c-KIT* mutational status and the MI.

## Supplementary Information

Below is the link to the electronic supplementary material.

Supplementary file1 (PDF 35 KB)

Supplementary file2 (DOCX 70.8 KB)

## Data Availability

Imaging and clinical research data are publicly available and can be found at https://xnat.bmia.nl/data/projects/worc, and are described in detail in https://doi.org/10.1101/2021.08.19.21262238.

## Code Availability

Programming code for the method presented in this study is available on Zenodo at https://doi.org/10.5281/zenodo.3839322.
