# Supplementary figures and images for: Differential Diagnosis and Molecular Stratification of Gastrointestinal Stromal Tumors on CT Images Using a Radiomics Approach
Source: J Digit Imaging. 2022 Jan 27;35(2):127–36. doi: 10.1007/s10278-022-00590-2 (PMC8921463; doi:10.1007/s10278-022-00590-2)

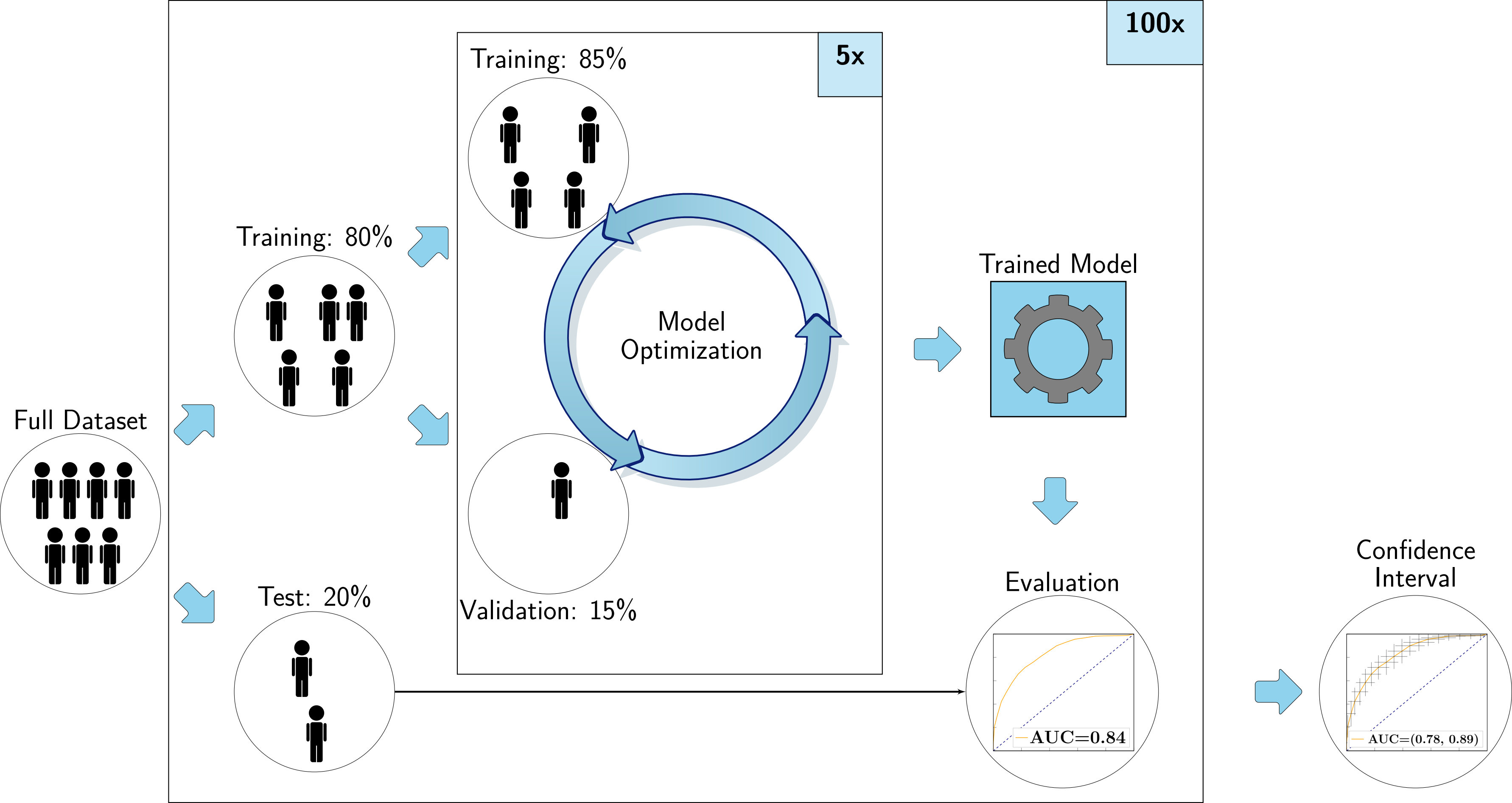

Supplement: Supplementary file 1 — Supplementary file1 (PDF 35 KB) [file 10278_2022_590_MOESM1_ESM.pdf]
