# Supplementary material for: Differential Diagnosis and Molecular Stratification of Gastrointestinal Stromal Tumors on CT Images Using a Radiomics Approach
Source: J Digit Imaging. 2022 Jan 27;35(2):127–36. doi: 10.1007/s10278-022-00590-2 (PMC8921463; doi:10.1007/s10278-022-00590-2)
Supplement: Supplementary file 2 — Supplementary file2 (DOCX 70.8 KB) [file 10278_2022_590_MOESM2_ESM.docx]

**Supplemental Materials**

**Supplemental Material 1: Radiomics feature extraction**

This supplemental material is similar to^1, 2^, but details relevant for the current study are highlighted.

A total of 564 radiomics features were used in this study. All features were extracted using the defaults for CT scans from the Workflow for Optimal Radiomics Classification (WORC)^3, 4^ toolbox (version 3.4.0), which internally uses the PREDICT^5^ (version 3.1.13) and PyRadiomics^6^ (version 3.0.1) feature extraction toolboxes. An overview of all features is depicted in **Supplemental Table S3**. For details on the mathematical formulation of the features, we refer the reader^7^. More details on the extracted features can be found in the documentation of the respective toolboxes, mainly the WORC documentation^8^. The code to extract the features has been published open-source^9^.

For CT scans, the images are by default not normalized as the scans already have a fixed unit and scale (i.e. Hounsfield), contrary to MRI. The images were not resampled, as this would result in interpolation errors. In PyRadiomics, a fixed bin count of 16 is used for the features requiring gray level discretization.

The features can be divided in several groups. Thirteen intensity features were extracted using the histogram of all intensity values within the ROIs and included several first-order statistics such as the mean, standard deviation and kurtosis. These describe the distribution of Hounsfield units within the lesion. Thirty-five shape features were extracted based only on the ROI, i.e. not using the image, and included shape descriptions such as the volume, compactness and circular variance. These describe the morphological properties of the lesion. Nine orientation features were used, describing the orientation of the ROI, i.e. not using the image. Lastly, 483 texture features were extracted using Gabor filters (144 features), Laplacian of Gaussian filters (36 features), vessel (i.e. tubular structures) filters (36 features)^10^, the Gray Level Co-occurrence Matrix (144 features)^7^, the Gray Level Size Zone Matrix (16 features)^7^, the Gray Level Run Length Matrix (16 features)^7^, the Gray Level Dependence Matrix (14 features)^7^, the Neighbourhood Grey Tone Difference Matrix (5 features)^7^, Local Binary Patterns (18 features)^11^, and local phase filters (36 features) ^12, 13^. These features describe more complex patterns within the lesion, such as heterogeneity, occurrence of blob-like structures, and presence of line patterns.

Most of the texture features include parameters to be set for the extraction. Beforehand, the values of the parameters that will result in features with the highest discriminative power for the classification at hand (e.g. GIST vs. non-GIST) are not known. Including these parameters in the workflow optimization, see **Supplemental Material 2**, would lead to repeated computation of the features, resulting in a redundant decrease in computation time. Therefore, alternatively, these features are extracted at a range of parameters as is default in WORC. The hypothesis is that the features with high discriminative power will be selected by the feature selection methods and/or the machine learning methods as described in **Supplemental Material 2**. The parameters used are described in **Supplemental Table S3**.

The dataset used in this study is heterogeneous in terms of acquisition protocols. Especially the variations in slice thickness may cause feature values to be dependent on the acquisition protocol. Hence, extracting robust 3D features may be hampered by these variations. To overcome this issue, all features were extracted per 2D axial slice and aggregated over all slices, which is default in WORC. Afterwards, several first-order statistics over the feature distributions were evaluated and used in the machine learning approach.

**Supplemental Material 2: Adaptive workflow optimization for automatic decision model creation**

This appendix is similar to ^1, 2^, but details relevant for the current study are highlighted.

The Workflow for Optimal Radiomics Classification (WORC) toolbox^3, 4^ (version 3.4.0), makes use of adaptive algorithm optimization to create the optimal performing workflow from a variety of methods. WORC defines a workflow as a sequential combination of algorithms and their respective parameters. To create a workflow, WORC includes algorithms to perform feature scaling, feature imputation, feature selection, oversampling, and machine learning. If used, as some of these steps are optional as described below, these methods are performed in the same order as described in this appendix. More details can be found in the WORC documentation^8^. The code to use WORC for creating the differential diagnosis and molecular analysis decision models in this specific study has been published open-source^9^.

Feature scaling was performed to make all features have the same scale, as otherwise the machine learning methods may focus only on those features with large values. This was done through z-scoring, i.e. subtracting the mean value followed by division by the standard deviation, for each individual feature. In this way, all features had a mean of zero and a variance of one. A robust version of z-scoring was used, in which outliers, i.e. values below the 5th percentile or above the 95th percentile, were excluded from computing the mean and variance.

When a feature could not be computed, e.g. a lesion was too small for a specific feature to be extracted or a division by zero occurred, feature imputation was used to estimate replacement values for the missing values. Strategies for imputation included 1) the mean; 2) the median; 3) the most frequent value; and 4) a nearest neighbor approach.

Feature selection was performed to eliminate features which were not useful to distinguish between the classes, e.g. GIST vs. non-GIST. These included;

1. a variance threshold, in which features with a low variance (<0.01) are removed. This method was always used, as this serves as a feature sanity check with almost zero risk of removing relevant features;
2. optionally, a group-wise search, in which specific groups of features (i.e. intensity, shape, and the subgroups of texture features as defined in **Supplemental Material 1**) are selected or deleted. To this end, each feature group had an on/off variable which is randomly activated or deactivated, which were all included as hyperparameters in the optimization;
3. optionally, the RELIEF method^14^, which ranks the features according to the differences between neighbouring samples. Features with larger differences between neighbours of different classes (i.e. GIST vs. non-GIST) are considered higher in rank;
4. optionally, selection using a machine learning model. Features are selected that are regarded important by a machine learning model trained on the dataset; hence the used model should be able to give the features an importance weight. Commonly used choices are LASSO, logistic regression, or a random forest;
5. optionally, individual feature selection through univariate testing. To this end, for each feature, a Mann-Whitney U test was performed to test for significant differences in distribution between the labels (e.g. GIST vs. non-GIST). Afterwards, only features with a p-value above a certain threshold were selected. A Mann-Whitney U test was chosen as features may not be normally distributed and the samples (i.e. patients) were independent; and 6) optionally, principal component analysis (PCA), in which either only those linear combinations of features were kept which explained 95% of the variance in the features or a limited number of components (between 10 – 50).

These feature selection methods may be combined by WORC, but only in the mentioned order.

Various resampling strategies can optionally be used, which can be used to overcome class imbalances and reduce overfitting on specific training samples. These included various methods from the imbalanced-learn toolbox^15^; random over-sampling, random under-sampling, near-miss resampling, the neighborhood cleaning rule, ADASYN, and SMOTE (regular, borderline, Tomek and the edited nearest neighbors variant).

Lastly, machine learning methods were used to determine a decision rule to distinguish the classes. These included; 1) logistic regression; 2) support vector machines; 3) random forests; 4) naive Bayes; and 5) linear discriminant analysis; 6) quadratic discriminant analysis; 7) AdaBoost ^16^; and 8) extreme gradient boosting ^17^.

Most of the included methods require specific settings or parameters to be set, which may have a large impact on the performance. As these parameters have to be determined before executing the workflow, these are so-called “hyperparameters”. In WORC, all parameters of all mentioned methods are treated as hyperparameters, since they may all influence the decision model creation. WORC simultaneously estimates which combination of algorithms and hyperparameters performs best. A comprehensive overview of all parameters is provided in the WORC documentation^8^.

By default in WORC, the performance is evaluated in a 100x random-split train-test cross-validation. In the training phase, a total of 1,000 pseudo-randomly generated workflows is created. These workflows are evaluated in a 5x random-split cross-validation on the training dataset, using 85% of the data for actual training and 15% for validation of the performance. All described methods are fit on the training datasets, and only tested on the validation datasets. The workflows are ranked from best to worst based on their mean performance on the validation sets using the weighted F1-score, which is the harmonic average of precision and recall. Due to the large number of workflows that is executed, there is a chance that the best performing workflow is overfitting, i.e. looking at too much detail or even noise in the training dataset. Hence, in each train-test cross-validation iteration, to create a more robust model and boost performance, WORC combines the 100 best performing workflows into a single final decision model, which is known as ensembling. These 100 best performing workflows are re-trained using the entire training dataset, and only tested on the test datasets. The ensemble is created through averaging of the probabilities, i.e. the chance of a patient being GIST or non-GIST, of these 100 workflows. This final ensemble model is almost certainly different in each of the 100x train-test random-split cross-validation iterations.

A full experiment consists of executing 500,000 workflows (1,000 pseudo-randomly generated workflows, times a 5x train-validation cross-validation times 100x train-test cross-validation), which can be parallelized. The computation time of training or testing a single workflow is on average less than a second, depending on the size of the dataset both in terms of samples (i.e. patients) and features. The largest experiment in this study, i.e. the differential diagnosis including 247 patients, had a computation time of approximately 12 hours on a 32 CPU core machine. The contribution of the feature extraction to the computation time was negligible.

**Supplemental Material 3: Robustness to segmentation and image acquisition variations**

Radiomics’ robustness to segmentation variations was assessed using the intra-class correlation coefficient (ICC) of the features on the subset of 30 GISTs which were segmented by two observers. “Good” and “excellent” reliability were defined by ICC > 0.75 and ICC > 0.90, respectively^18^. Moreover, the impact of ICC-based feature selection on model performance was assessed by creating models using only features with good or excellent reliability.

Robustness to variations in the acquisition parameters was assessed by using ComBat harmonization^19, 20^. In ComBat, feature distributions are harmonized for variations in the imaging acquisition, e.g. due to differences in hospitals, manufacturers, or acquisition parameters. When dividing the dataset into groups based on these variations, the groups have to remain sufficiently large to estimate the harmonization parameters. In our study, groups were defined based on manufacturer alone, or based on protocol, defined as the combination of manufacturer and slice thickness (above or below the median). No moderation variable was used.

**Supplemental References**

1. Vos M, Starmans MPA, Timbergen MJM, et al. Radiomics approach to distinguish between well differentiated liposarcomas and lipomas on MRI. *The British Journal of Surgery*. 2019-12 2019;106(13):1800-1809. doi:10.1002/bjs.11410

2. Timbergen MJM, Starmans MPA, Padmos GA, et al. Differential diagnosis and mutation stratification of desmoid-type fibromatosis on MRI using radiomics. *European Journal of Radiology*. 2020/09/08/ 2020:109266. doi:10.1016/j.ejrad.2020.109266

3. Starmans MPA, Van der Voort SR, Phil T, Klein S. Workflow for Optimal Radiomics Classification (WORC). Zenodo. Accessed 22-12-2021, <https://github.com/MStarmans91/WORC>. doi:10.5281/zenodo.3840534

4. Starmans MPA, van der Voort SR, Phil T, et al. Reproducible radiomics through automated machine learning validated on twelve clinical applications. *arXiv:210808618*. 2021;doi:arXiv:2108.08618

5. van der Voort SR, Starmans MPA. Predict: a Radiomics Extensive Digital Interchangable Classification Toolkit (PREDICT). Zenodo. Accessed 25-02-2021, <https://github.com/Svdvoort/PREDICTFastr>. doi:10.5281/zenodo.3854839

6. Van Griethuysen JJ, Fedorov A, Parmar C, et al. Computational radiomics system to decode the radiographic phenotype. *Cancer research*. 2017;77(21):e104-e107. doi:10.1158/0008-5472.CAN-17-0339

7. Zwanenburg A, Vallières M, Abdalah M, et al. The Image Biomarker Standardization Initiative: Standardized Quantitative Radiomics for High-Throughput Image-based Phenotyping. *Radiology*. 03/10 2020;295:191145. doi:10.1148/radiol.2020191145

8. Starmans MPA. Workflow for Optimal Radiomics Classification (WORC) Documentation. Zenodo. Accessed 25-02-2021, <https://worc.readthedocs.io>. doi:10.5281/zenodo.3840534

9. Starmans MPA. GISTRadiomics. Zenodo. Accessed 22-12-2021, <https://github.com/MStarmans91/GISTRadiomics>. doi:10.5281/zenodo.3839323

10. Frangi AF, Niessen WJ, Vincken KL, Viergever MA. Multiscale vessel enhancement filtering. Springer Berlin Heidelberg; 1998:130-137. doi:10.1007/BFb0056195

11. Ojala T, Pietikainen M, Maenpaa T. Multiresolution gray-scale and rotation invariant texture classification with local binary patterns. *IEEE Transactions on Pattern Analysis and Machine Intelligence*. 2002;24(7):971-987. doi:10.1109/TPAMI.2002.1017623

12. Kovesi P. Phase congruency detects corners and edges. In: *The Australian pattern recognition society conference: DICTA*. 2003:

13. Kovesi P. Symmetry and asymmetry from local phase. In: *Tenth Australian joint conference on artificial intelligence*. Citeseer; 1997:2–4.

14. Urbanowicz RJ, Olson RS, Schmitt P, Meeker M, Moore JH. Benchmarking relief-based feature selection methods for bioinformatics data mining. *Journal of Biomedical Informatics*. September 1, 2018 2018;85:168-188. doi:10.1016/j.jbi.2018.07.015

15. Lemaitre G, Nogueira F, Aridas CK. Imbalanced-learn: A Python Toolbox to Tackle the Curse of Imbalanced Datasets in Machine Learning. *Journal of Machine Learning Research*. 2017;18

16. Freund Y, Schapire RE. A Decision-Theoretic Generalization of On-Line Learning and an Application to Boosting. *Journal of Computer and System Sciences*. 1997/08/01/ 1997;55(1):119-139. doi:10.1006/jcss.1997.1504

17. Chen T, He T, Benesty M, Khotilovich V, Tang Y. Xgboost: extreme gradient boosting. *R package version 04-2*. 2015:1-4.

18. Koo TK, Li MY. A Guideline of Selecting and Reporting Intraclass Correlation Coefficients for Reliability Research. *Journal of Chiropractic Medicine*. 2016/06/01/ 2016;15(2):155-163. doi:10.1016/j.jcm.2016.02.012

19. Fortin J-P, Parker D, Tunç B, et al. Harmonization of multi-site diffusion tensor imaging data. *NeuroImage*. 2017/11/01/ 2017;161:149-170. doi:10.1016/j.neuroimage.2017.08.047

20. Orlhac F, Boughdad S, Philippe C, et al. A Postreconstruction Harmonization Method for Multicenter Radiomic Studies in PET. *Journal of Nuclear Medicine*. 08/2018 2018;59(8):1321-1328. doi:10.2967/jnumed.117.199935

**Supplemental Table S1.** P-values of features from univariate tests between GIST and non-GIST patients after Bonferroni correction. A Mann-Whitney U test was used for continuous variables, a Chi-square test for categorical variables. Only features with a p-value < 0.05, which are considered statistically significant, are shown. Besides the feature names, several of the feature labels also include the parameters used. More details on the features can be found in Supplemental Materials 1.

| **Label** | **Mann-Whitney U P** | **Chi2 P** |
| --- | --- | --- |
| semf_location |  | 1,14E-17 |
| of_COM_x | 7,46E-08 |  |
| hf_energy | 1,59E-05 |  |
| of_COM_Index_x | 3,06E-05 |  |
| tf_Gabor_mean_F0.2_A0.79 | 0,000 |  |
| tf_GLRLM_LongRunEmphasis | 0,001 |  |
| tf_GLRLM_RunVariance | 0,001 |  |
| tf_GLSZM_ZonePercentage | 0,001 |  |
| tf_GLRLM_ShortRunEmphasis | 0,001 |  |
| tf_GLRLM_RunPercentage | 0,001 |  |
| tf_GLRLM_RunLengthNonUniformityNormalized | 0,001 |  |
| tf_GLDM_DependenceVariance | 0,001 |  |
| tf_Gabor_mean_F0.2_A2.36 | 0,002 |  |
| tf_GLDM_LargeDependenceEmphasis | 0,002 |  |
| tf_GLDM_SmallDependenceLowGrayLevelEmphasis | 0,003 |  |
| tf_GLCMMS_homogeneityd3.0A0.79mean | 0,004 |  |
| tf_Gabor_energy_F0.2_A0.79 | 0,005 |  |
| tf_GLRLM_LongRunHighGrayLevelEmphasis | 0,006 |  |
| tf_GLDM_SmallDependenceEmphasis | 0,006 |  |
| tf_Gabor_energy_F0.2_A2.36 | 0,007 |  |
| sf_area_std_2D | 0,007 |  |
| tf_GLDM_DependenceNonUniformityNormalized | 0,008 |  |
| tf_GLDM_LargeDependenceLowGrayLevelEmphasis | 0,009 |  |
| tf_Gabor_energy_F0.2_A1.57 | 0,009 |  |
| tf_GLDM_LargeDependenceHighGrayLevelEmphasis | 0,010 |  |
| tf_Gabor_mean_F0.2_A0.0 | 0,011 |  |
| tf_GLCMMS_homogeneityd3.0A0.0mean | 0,013 |  |
| sf_area_max_2D | 0,015 |  |
| tf_GLSZM_LargeAreaHighGrayLevelEmphasis | 0,016 |  |
| tf_GLSZM_LargeAreaEmphasis | 0,016 |  |
| tf_GLSZM_ZoneVariance | 0,016 |  |
| tf_Gabor_energy_F0.2_A0.0 | 0,017 |  |
| hf_min | 0,017 |  |
| sf_area_avg_2D | 0,022 |  |
| tf_GLRLM_LongRunLowGrayLevelEmphasis | 0,024 |  |
| of_COM_y | 0,025 |  |
| tf_GLSZM_LargeAreaLowGrayLevelEmphasis | 0,027 |  |
| tf_Gabor_median_F0.05_A2.36 | 0,027 |  |
| sf_shape_Maximum2DDiameterSlice | 0,031 |  |
| tf_GLRLM_GrayLevelNonUniformityNormalized | 0,039 |  |
| vf_Frangi_inner_energy_SR(1.0. 10.0)_SS2.0 | 0,039 |  |
| tf_Gabor_mean_F0.5_A2.36 | 0,045 |  |
| tf_Gabor_kurtosis_F0.05_A0.79 | 0,046 |  |

*Abbreviations: GLCM: gray level co-occurrence matrix; GLCMMS: GLCM multislice; NGTDM: neighborhood gray tone difference matrix; GLSZM: gray level size zone matrix; GLRLM: gray level run length matrix; LBP: local binary patterns; LoG: Laplacian of Gaussian; std: standard deviation.

**Supplemental Table S2.** Performance of the radiomics models for the differential diagnosis based on imaging using only features with good (ICC > 0.75) or excellent (ICC > 0.90) reliability; and using ComBat harmonization per manufacturer or per protocol (manufacturer and high/low slice thickness). For each metric, the mean and 95% confidence interval over the 100x random-split cross-validation iterations are given.

|  | ICC > 0.75 | ICC > 0.90 | ComBat Manufacturer | ComBat Protocol |
| --- | --- | --- | --- | --- |
| **AUC** | 0.67 [0.60, 0.75] | 0.67 [0.60, 0.74] | 0.80 [0.74, 0.86] | 0.77 [0.71, 0.83] |
| **BCA** | 0.64 [0.57, 0.71] | 0.62 [0.56, 0.68] | 0.73 [0.67, 0.79] | 0.70 [0.64, 0.76] |
| **Sensitivity** | 0.50 [0.40. 0.60] | 0.50 [0.40, 0.60] | 0.72 [0.61, 0.83] | 0.69 [0.59, 0.80] |
| **Specificity** | 0.74 [0.63, 0.85] | 0.74 [0.63, 0.85] | 0.74 [0.64, 0.84] | 0.71 [0.61, 0.80] |

* Abbreviations: AUC: area under the receiver operating characteristic curve; BCA: balanced classification accuracy.

**Supplemental Table S3.** Overview of the 564 features used in this study. GLCM features were calculated in four different directions (0, 45, 90, 135 degrees) using 16 gray levels and pixel distances of 1 and 3. LBP features were calculated using the following three parameter combinations: 1 pixel radius and 8 neighbours, 2 pixel radius and 12 neighbours, and 3 pixel radius and 16 neighbours. Gabor features were calculated using three different frequencies (0.05, 0.2, 0.5) and four different angles (0, 45, 90, 135 degrees). LoG features were calculated using three different widths of the Gaussian (1, 5 and 10 pixels). Vessel features were calculated using the full mask, the edge, and the inner region. Local phase features were calculated on the monogenic phase, phase congruency and phase symmetry.

| Histogram  (13 features) | LoG  (13*3=39 features) | | Vessel  (12*3=39 features) | GLCM (MS)  (6*3*4*2=144 features) | | Gabor  (13*4*3=156 features) | NGTDM  (5 features) | LBP  (13*3=39 features) |  |
| --- | --- | --- | --- | --- | --- | --- | --- | --- | --- |
| min  max  mean  median  std  skewness  kurtosis  peak  peak position  range  energy  quartile range  entropy | min  max  mean  median  std  skewness  kurtosis  peak  peak position  range  energy  quartile  entropy | | min  max  mean  median  std  skewness  kurtosis  peak  peak position  range  energy  quartile  entropy | contrast (normal, MS mean + std)  dissimilarity (normal, MS mean + std)  homogeneity(normal, MS mean + std)  angular second moment (ASM) (normal, MS mean + std)  energy (normal, MS mean + std)  correlation (normal, MS mean + std) | | min  max  mean  median  std  skewness  kurtosis  peak  peak position  range  energy  quartile range  entropy | busyness  coarseness  complexity  contrast  strength | min  max  mean  median  std  skewness  kurtosis  peak  peak position  range  energy  quartile range  entropy |  |
| GLSZM  (16 features) | | **GLRM**  **(16 features)** | | | **GLDM**  **(14 features)** | **Shape**  **(35 features)** | **Orientation**  **(9 features)** | **Local phase**  **(13*3=39 features)** |  |
| Gray Level Non Uniformity  Gray Level Non Uniformity Normalized  Gray Level Variance  High Gray Level Zone Emphasis  Large Area Emphasis  Large Area High Gray Level Emphasis  Large Area Low Gray Level Emphasis  Low Gray Level Zone Emphasis  SizeZoneNonUniformity  SizeZoneNonUniformityNormalized  SmallAreaEmphasis  SmallAreaHighGrayLevelEmphasis  SmallAreaLowGrayLevelEmphasis  ZoneEntropy  ZonePercentage  ZoneVariance | | Gray Level Non Uniformity  Gray Level Non Uniformity Normalized  Gray Level Variance  High Gray Level Run Emphasis  Long Run Emphasis  Long Run High Gray Level Emphasis  Long Run Low Gray Level Emphasis  Low Gray Level Run Emphasis  RunEntropy  RunLengthNonUniformity  RunLengthNonUniformityNormalized  RunPercentage  RunVariance  ShortRunEmphasis  ShortRunHighGrayLevelEmphasis  ShortRunLowGrayLevelEmphasis | | | Dependence Entropy  Dependence Non-Uniformity  Dependence Non-Uniformity Normalized  Dependence Variance  Gray Level Non-Uniformity  Gray Level Variance  High Gray Level Emphasis  Large Dependence Emphasis  Large Dependence High Gray Level Emphasis  Large Dependence Low Gray Level Emphasis  Low Gray Level Emphasis  Small Dependence Emphasis  Small Dependence High Gray Level Emphasis  Small Dependence Low Gray Level Emphasis | compactness (mean + std)  radial distance (mean + std)  roughness (mean + std)  convexity (mean + std)  circular variance (mean + std)  principal axes ratio (mean + std)  elliptic variance (mean + std)  solidity (mean + std)  area (mean, std, min + max  volume (total, mesh, volume)  elongation  flatness  least axis length  major axis length  minor axis length  maximum diameter 3D  maximum diameter 2D (rows, columns, slices)  sphericity  surface area  surface volume ratio | theta_x  theta_y  theta_z  COM index x  COM index y  COM index z  COM x  COM y  COM z | min  max  mean  median  std  skewness  kurtosis  peak  peak position  range  energy  quartile  entropy |  |

*Abbreviations: COM: center of mass; GLCM: gray level co-occurrence matrix; MS: multi slice; NGTDM: neighborhood gray tone difference matrix; GLSZM: gray level size zone matrix; GLRLM: gray level run length matrix; LBP: local binary patterns; LoG: Laplacian of Gaussian; std: standard deviation.
